# Supplementary material for: Conditional chemoconnectomics (cCCTomics) as a strategy for efficient and conditional targeting of chemical transmission
Source: eLife. 2024 Apr 30;12:RP91927. doi: 10.7554/eLife.91927 (PMC11060718; doi:10.7554/eLife.91927)
Supplement: Figure 3—source data 3. [file elife-91927-fig3-data3.zip › Figure 3 source data3 part 2.zip!/Figure 3-Source data 3-part2/1002-9-CRISPResso_Report_e53dd8m5.zip!/CRISPResso_on_1002_10/9.Alleles_frequency_table_around_sgRNA_gtatctgtccagctacgtga.pdf]

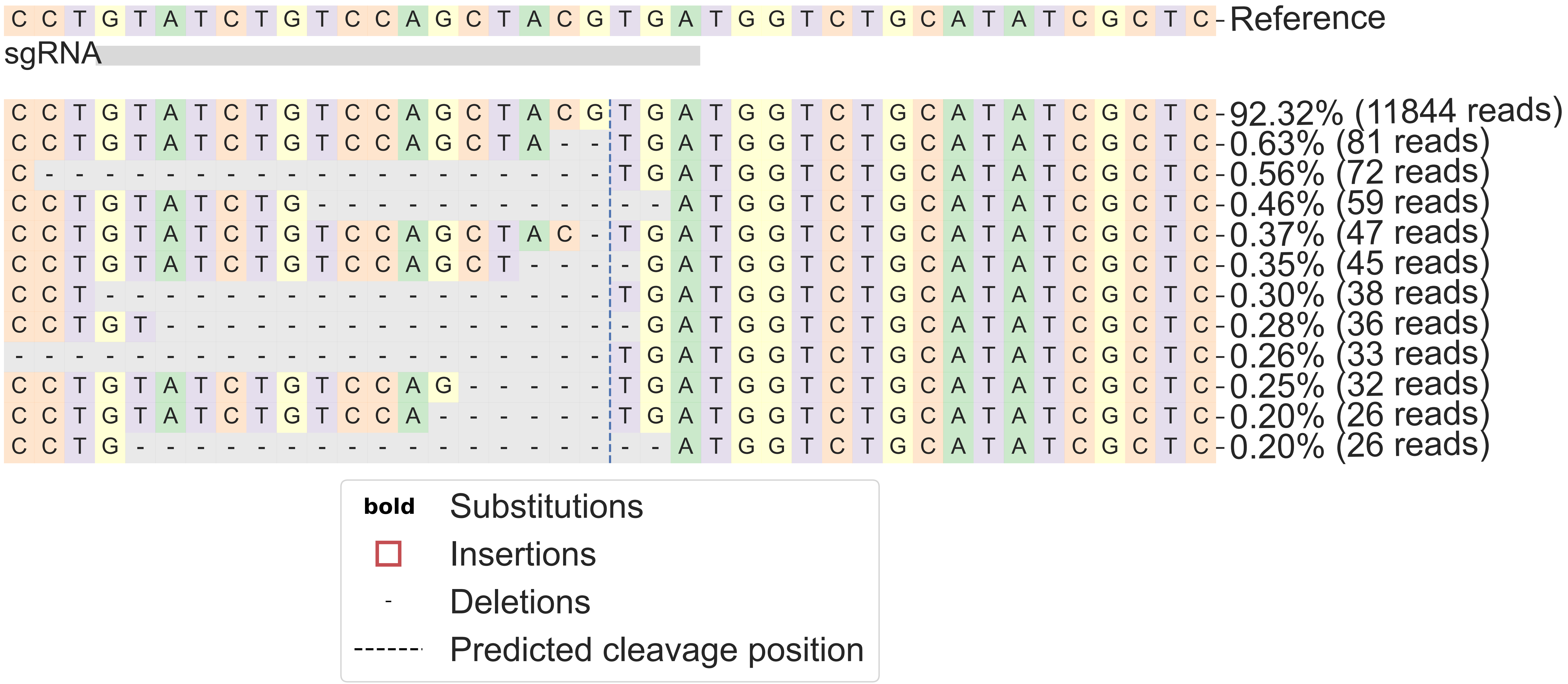
CCTGTATCTGTCCAGCTACGTGATGGTCTGCATATCGCTC Reference
sgRNA
CCTGTATCTGTCCAGCTACGTGATGGTCTGCATATCGCTC 92.32% (11844 reads)
CCTGTATCTGTCCAGCTA--TGATGGTCTGCATATCGCTC 0.63% (81 reads)
C-------------------TGATGGTCTGCATATCGCTC 0.56% (72 reads)
CCTGTATCTG------------ATGGTCTGCATATCGCTC 0.46% (59 reads)
CCTGTATCTGTCCAGCTAC-TGATGGTCTGCATATCGCTC 0.37% (47 reads)
CCTGTATCTGTCCAGCT----GATGGTCTGCATATCGCTC 0.35% (45 reads)
CCT-----------------TGATGGTCTGCATATCGCTC 0.30% (38 reads)
CCTGT----------------GATGGTCTGCATATCGCTC 0.28% (36 reads)
--------------------TGATGGTCTGCATATCGCTC 0.26% (33 reads)
CCTGTATCTGTCCAG-----TGATGGTCTGCATATCGCTC 0.25% (32 reads)
CCTGTATCTGTCCA------TGATGGTCTGCATATCGCTC 0.20% (26 reads)
CCTG------------------ATGGTCTGCATATCGCTC 0.20% (26 reads)
bold Substitutions
Insertions
- Deletions
------ Predicted cleavage position
